# Burnout and dropout intention in medical students: the protective role of academic engagement

**DOI:** 10.1186/s12909-021-03094-9

**Published:** 2022-02-07

**Authors:** Sara Calcatin, Jorge Sinval, Lia Lucas Neto, João Marôco, António Gonçalves Ferreira, Pedro Oliveira

**Affiliations:** 1 Faculdade de Medicina, Universidade de Lisboa, Lisbon, Portugal; 2 Business Research Unit (BRU-IUL), Instituto Universitário de Lisboa (ISCTE-IUL), Lisbon, Portugal; 3 William James Center for Research, ISPA - Instituto Universitário, Lisbon, Portugal; 4 Faculty of Philosophy, Sciences and Languages of Ribeirão Preto, University of São Paulo, Ribeirão Preto - SP, Brazil; 5 CiiEM Centro investigação interdisciplinar Egas Moniz, Almada, Portugal

**Keywords:** Student burnout, Academic engagement, Coping, Medical students, Dropout intention

## Abstract

**Introduction:**

The influence of burnout, academic engagement, and their interaction in dropout intention among medical students should be further studied. Current research shows its consequences are relevant, however, there is little understanding on burnout and academic engagement moderation in dropout intention. The current study tested a model that relates the effects of coping strategies, social support satisfaction, general distress on academic engagement, burnout, and dropout intention, on medical students.

**Methods:**

Through an online survey a non-probabilistic sample of one Medical Faculty's 1st- and 2nd-year students was recruited. Cross-sectional data were collected using psychometric instruments (Maslach Burnout Inventory – Student Survey, Social Support Satisfaction Scale for College Students, Brief COPE Scale for College Students, University Student Engagement Inventory, and Depression, Anxiety and Stress Scale), sociodemographic and academic variables, and analyzed using structural equation modeling.

**Results:**

532 students (76% response rate) enrolled in the study. Latent variables structural model presented a satisfactory fit to the data and confirmed the expected negative path between burnout and dropout intention (β_*DI<-SB*_=0.430; *p*<.001) and the latent moderation burnout x engagement (β_DI<-SB*SE_=-0.218; *p*<.001).

**Conclusion:**

Academic engagement attenuates the impact of burnout on dropout intention, working as a protective factor. Social support satisfaction and adaptive coping are associated with increased levels of academic engagement, and general distress and maladaptive coping are associated with burnout. Medical Schools should develop interventions to prevent dropout intention, tackle students' stress and academic challenges, and develop their academic engagement levels.

## Introduction

Student burnout is the triad of exhaustion, cynicism, and feelings of inefficacy [1]. Exhaustion refers to feelings of exceeding emotional resources due to the requirements of the study. Cynicism is a negative, insensitive, or excessively detached response to the study, colleagues, teachers, and patients. Feelings of inefficacy mean reduced academic achievement as a decline in feelings of competence and achievement as a student. A systematic review of 2021 on the prevalence of burnout syndrome in university students showed estimates of 55.4% for emotional exhaustion, 31.6% for cynicism and 30.9% for academic efficacy. Also, it mentioned that medical students had higher burnout prevalence in contrast with other courses [3]. Medical students tend to experience student burnout at some point during graduation and later as physicians [2]. The personal, psychological, and financial consequences of burnout and dropout in medical students have shown to be relevant, such as the emotional suffering and health problems, and the waste of time, resources and money [4].

Burnout is associated with general distress, poor educational performance, college dropout, suicidal ideation [5] and substance use [6], mainly during medical school [2]. Although depression and burnout overlap to some extent, their correlation strength is not yet fully understood [7]. Occupational stress and anxiety symptoms can be directly related to burnout, which means the higher the stress and anxiety, the higher the burnout level [7].

College dropout intention has a wide range of negative consequences on the individual, family, faculty, and community [4]. For instance, dropout intention is related to an individual’s expectations not being met, symptoms of loss and frustration, and financial costs [4]. European Commission’s Education and Training Monitor 2020 showed that 10.2% of students are early leavers from education and training [8]. The Portuguese context is not an exception in terms of higher than desirable dropout rates [4]. Previous research found that 11% of medical students had serious thoughts of dropping out of medical school each year [9]. In the USA, resident trainees’ second leading cause of death is suicide (4.1 per each 100,000), and approximately 10 out of 100 medical students report suicidal ideation [10, 11]. 

Some of the causes of medical student burnout are known – high academic requirements, demanding nature of the contents, heavy workload, stress of the exams [12], however there is an insufficient number of studies on predictive psychological variables of burnout in medical students.

In this regard, there is a need to understand better how student burnout, and academic engagement relate to dropout intention [5]. Academic engagement outlines student disposition to engage in school activities [13]. Behavioral, emotional, and cognitive engagement describe academic engagement dimensions [5]. Behavioral academic engagement encompasses student conduct and participation in classroom tasks and extracurricular activities [5]. Emotional academic engagement refers to student willingness to invest the necessary efforts to comprehend and master each subject [5]. Cognitive academic engagement concerns feelings of school belonging, beliefs about the value of schooling, and attention to teacher’s instructions [5]. 

Another variable of interest regarding burnout is coping [14]. Carver et al. [15] described 14 coping mechanisms. One possible classification of coping strategies splits them into adaptive coping and maladaptive coping. Adaptive coping strategies are seeking help, information, social support, accepting, planning, and reframing problems with faith or humor [5]. Adaptive coping is associated with academic engagement [5]. In contrast, non-adaptive coping strategies encompass self-distraction, disengagement, self-blame, denial, and substance use [5]. Non-adaptive coping is associated with burnout [5]. Student burnout and general distress (i.e. anxiety, depression, and stress) are negatively associated with adaptative coping strategies as spend time with family and friends [16]. Not all coping strategies are expected to be equally effective in stress management [17], some coping strategies can be protective to health (adaptive) while maladaptive strategies are detrimental to health. Being expected, that academic engagement shows a positive association with adaptive coping and a negative relation with maladaptive coping, while student burnout is expected to have the opposite associations (i.e. positively associated with maladaptive strategies, and negatively related with adaptive coping) [18].

Additionally, social support is a key factor of study. Social support satisfaction is the set of information that one experiences that make him/her believe that he/she is supported, loved, and valued in a communication network and mutual obligations [19]. Social support is expected to be negatively related to burnout. This association was evident in a meta-analytic study with more than 95,000 students, where support from school or teacher have the strongest negative relationship to student burnout [20]. Social support showed to be central to student engagement in academic activities [21] being expected to be positively related to the satisfaction with social support.

**Fig. 1:**
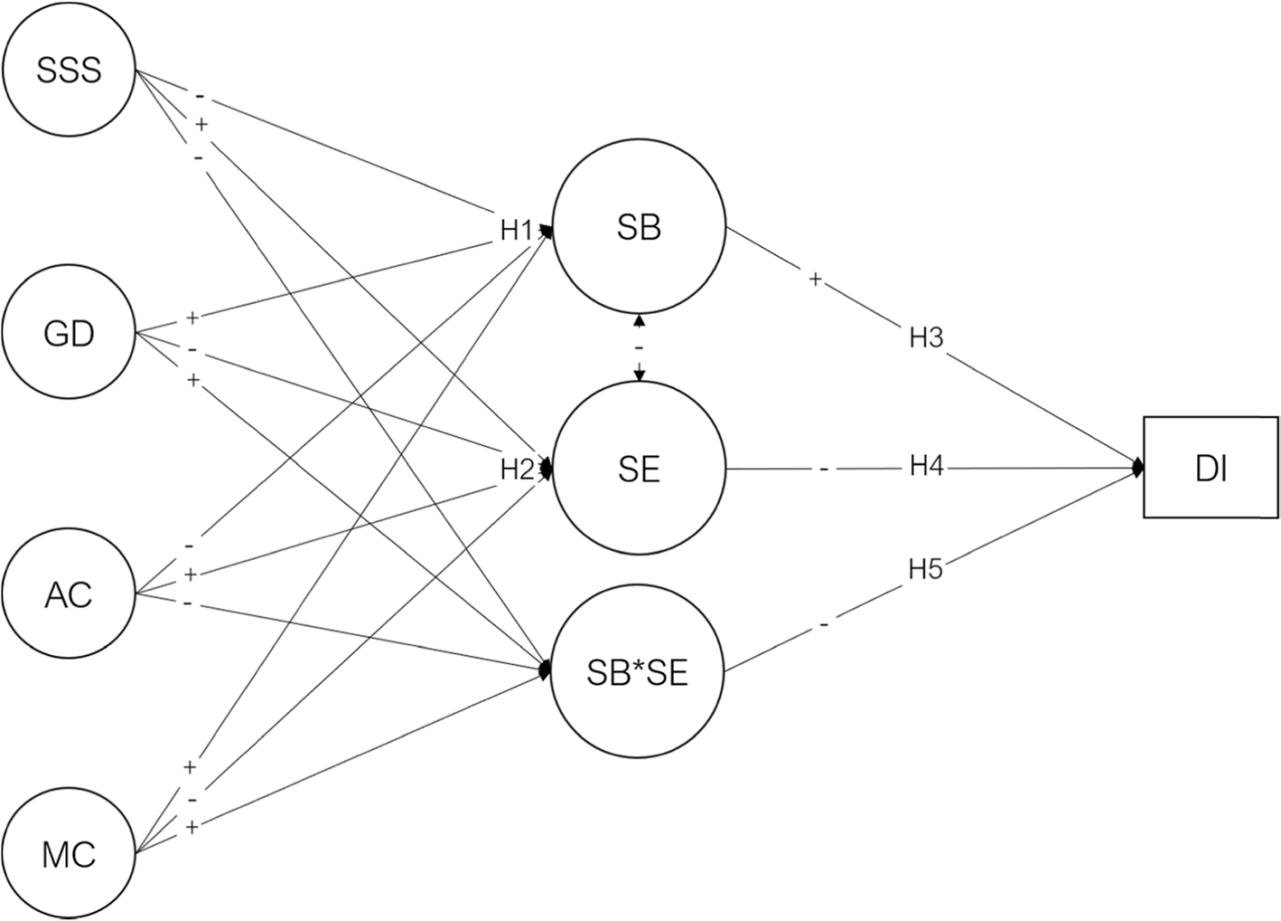
Student burnout and engagement model. SSS=Satisfaction with Social Support, GD=General Distress, AC=Adaptive Coping, MC=Maladaptive Coping, SB=Student Burnout, SE=Academic Engagement, SB*SE=Student Burnout interaction with Academic Engagement, DI=Dropout Intention. Plus sign (+) represents positive expected effect and minus sign (-) represents negative expected effect

Therefore, this study aims to add to the medical education literature that satisfaction with social support, adaptive coping mechanisms, and academic engagement are answers to burnout and dropout intention conundrum in medical students. In general, the hypotheses established in this study are presented in the diagram (Fig. 1), which was tested in a sample of medical students to determine potential predictors of student burnout and academic engagement that may be intervened and prevent dropout.

### Research Hypotheses

As academic workload and high standards in medical schools are not likely an object of change, it is crucial to understand the psychological factors underlying this phenomenon to tackle student burnout [12] and dropout intention [8]. This paper aims to test a model that posits psychological variables (coping, satisfaction with social support, and general distress) as predictors of burnout, academic engagement, and its interaction, which predict dropout intention. Hypothesis 1 states that social support satisfaction, adaptive and maladaptive coping, and general distress are significant predictors of student burnout (H1) and while hypotesis 2 states that those predictors impact also academic engagement (H2). Moreover, hypothesis 3 assumes that burnout is a risk factor for dropout intention (H3). In contrast, hypothesis 4 states that academic engagement is a protective factor to dropout intention (H4). Finally, hypothesis 5 assumes that t he latent interaction between academic engagement and burnout is a significant predictor of dropout intention (H5). Figure 1 depicts the research hypotheses.

## Methods

### Procedures

 The Ethics Committee of CHLN-HSM and Faculty of Medicine of the University of Lisbon (FMUL) approved this study (Ref.N.210/20). An online survey was comprised of psychometric scales and a social demographic questionnaire through *LimeSurvey* software [22]. The electronic informed consent was presented first, and has been accepted by all the participants, allowing them to proceed further in the questionnaire. All participants could leave the study at any time.

With the intent to improve answers’ quality, a 1st-year class of 28 medical students enrolled in a pilot study to evaluate the platform used and items’ comprehensibility. The 95% trimmed mean of the pilot’s filling time was 12 min and 59 s. These students sent global comments to the research team with feedback about the survey and ideas to improve response rate. Pilot study data are available from the corresponding author on reasonable request.

Afterwards, it was took into consideration that it is essential to carefully choose and justify the data collecting moment of the academic year. For example, proximity to exams, both before and after, can seriously affect the psychological variables considered. Therefore, the data of the main study was collected for ten days, two weeks after classes had started. All potential participants received an invitation on the same day and two reminders during the next few days. Everyone received an automatic report about their answers and their comparison to the overview.

### Participants

A minimum sample of 500 observations was defined as recommended by Hair et al. [23] for models with large number of constructs. The sample included students from the 1st or 2nd-year of the 2020-21 academic year of FMUL. Non-medical students and medical students from other years did not participate. A total of 702 invitations to participate in the study were sent (pilot sample included).

### Measures

Online self-report psychometric instruments were used together with sociodemographic and academic questions. Each instrument was chosen considering its psychometric properties, frequency of use with higher education students, and cost of use. All instruments had already their adapted versions for Portuguese college students published.

#### Maslach Burnout Inventory – Student Survey (MBI-SSi)

The Maslach Burnout Inventory — Student Survey with the efficacy dimension reversed (MBI-SSi) was selected to measure students’ burnout levels.[24] MBI-SSi has 15 items (0 “Never/Not once” to 6 “Always/Every day”). A second-order latent factor, *burnout*, with three first-order dimensions was used (i.e., emotional exhaustion, cynicism, and inefficacy). A sample item is “I feel used up at the end of a day at university” (Exhaustion).

#### University Student Engagement Inventory (USEI)

The Portuguese version of the University Student Engagement Inventory (USEI) was used to measure students’ academic engagement as a second-order factor with three first-order dimensions: emotional, cognitive, and behavioral [25]. It has 15 items (1 “Never” to 5 “Always”). A sample item was “I usually participate actively in group assignments” (Behavioral Engagement).

#### Social Support Satisfaction Scale for College Students (SSSS)

The Portuguese version of the Social Support Satisfaction Scale (SSSS) for College Students was used to measure students’ satisfaction with their social support as a second-order factor with four first-order dimensions: social activities, satisfaction with family, satisfaction with friendship, and intimacy [19] The SSSS has 12 items (1 “Strongly disagree” to 5 “Strongly agree”). A sample item is “I am satisfied with the number of friends I have” (Satisfaction with Friendship).

#### Brief-COPE Scale for College Students (Brief-COPE)

The Portuguese version of the Brief-COPE Scale for College Students (Brief-COPE) was selected to measure students coping strategies [26]. It operationalizes 14 coping dimensions which had two second-order factors [27]: adaptive coping strategies (Active Coping, Planning, Use of Instrumental Support, Use of Emotional Support, Religion, Positive Reframing, Acceptance, Humor) and maladaptive coping strategies (Denial, Venting, Behavioral Disengagement, Substance Use, Self-Distraction, Self-Blame). This instrument has 28-items (0 “I never do this” to 4 “I always do this”). A sample item is “I’ve been concentrating my efforts on doing something about the situation I’m in” (Active Coping).

#### Depression, Anxiety and Stress Scale (DASS-21)

The Depression, Anxiety and Stress Scale (DASS-21) Portuguese version was applied to measure students’ general distress as a second-order factor [28]. The 21 items (0 “Did not apply to me at all” to 3 “Applied to me very much, or most of the time”) comprise three first-order dimensions: Depression, Anxiety, and Stress. A sample item is “I couldn’t seem to experience any positive feeling at all” (Depression).

#### Dropout intention

Students answered if they had thought about dropping out of medical school with a dichotomic scale (“Yes” or “No”); i.e. “Have you ever thought about dropping out of medical school?” The selection of this item intends to filter students who considered the possibility of dropping out from medical school independently of the level of seriousness of the thoughts.

#### Sociodemographic questions

Students answered about their sex, age, and administrative region of origin.

### Data analysis

The data analysis was conducted using the R programming language [29] through the graphical user interface, *RStudio* [30]. The *skimr* package [31] was used to obtain some of the descriptive statistics (mean, standard deviation, minimum value, 25th percentile, median, 75th percentile, maximum value) and the histogram for each of the instruments’ items. Other descriptive statistics were calculated: the coefficient of variation (CV) through the *sjstats* package [32], the standard error of the mean (SEM) through the *plotrix* package [33], and the mode through the *modeest* package [34]. The skewness using the “sample” method, and the kurtosis using the “sample excess” method, were calculated using the *PerformanceAnalytics* package [35]. Severe univariate normality violations were assumed for absolute values of |*sk*| >3 and |*ku*| >7 [36, 37].

The confirmatory factor analysis (CFA) was used to assess if the collected data confirmed the expected dimensionality of the used instruments. All SEM analysis were done with the *lavaan* package [38] using the weighted least squares means and variances (WLSMV) estimation method [39]. The *TLI* (Tucker Lewis Index), the *SRMR* (Standardized Root Mean Square Residual), the *RMSEA* (Root Mean Square Error of Approximation), *NFI* (Normed Fit Index), χ^2^/*df* (ratio chi-square and degrees of freedom), and *CFI* (Comparative Fit Index) were used as goodness-of-fit indices. The fit of the models was considered good if χ^2^/*df* < 5, values of *SRMR* and *RMSEA* < .08, values of *CFI*, *NFI,* and *TLI* > .95. The reliability of the scores was assessed based on the estimates of internal consistency. The ω based on the polychoric correlation matrices were calculated for first-order factors [40, 41]. For second-order factors, the variance of the first-order factors explained by the second-order factor (ω_*L2*_), the proportion of variance explained by second-order factor after partialling the uniqueness of the first-order factor (ω_*partial L1*_), the proportion of the second-order factor explaining the total score (ω_*L1*_) were calculated. All the internal consistency estimates were obtained through the *semTools* package [42].

The structural model was analyzed through the structural equation modeling technique using the *lavaan* package [38] implemented through a two-step approach [37]. The latent interaction between student burnout and academic engagement was operationalized with the *semTools* package [42] using double-mean centering for the product of the indicators [43] and a match-paired approach [44]. Confidence intervals (95%) were provided for all paths. The same criteria established in the evaluation of the measurement models (i.e. CFA) were used to assess the goodness-of-fit of the latent variable structural model. For all statistical tests, α = .05 was used.

## Results

This cross-sectional, observational study analyzed 532 medical students of FMUL, 417 were complete (*n*_*1st year*_=215; *n*_*2nd year*_=174; *n*_*pilot*_=28). Most students were females (71%), with a mean age of 19.48-years-old (*SD*=2.75), 53% from the Lisbon or Setubal districts. The response rate was 76%. About 78% of participants completely answered. The 95% trimmed meantime of response was 12 min and 46 s.

### Measurement Model

#### Validity Evidence based on the Internal Structure

For each instrument, the item’s distributional properties were inspected. Then, the dimensionality and the reliability of the scores in terms of internal consistency for first- and second-order factors were analyzed.

##### Items’ Distributional Properties

As shown in Table 1, all items presented skewness and kurtosis values that were not indicative of severe univariate normality violations [36, 37], except two items (Item 25 and Item 26) of Brief COPE, both from the substance use dimension. Those two items were the only items from Brief COPE that did not present the full range of possible answers, as shown in the histograms of Table 1. All items of the MBI-SSi, DASS-21 and SSSS had the full range of possible answers, while the USEI did not have the maximum possible range of answers in 10 items (i.e., items 1, 2, 3, 5, 7, 8, 9, 13, 14, 15). 

**Table 1:**
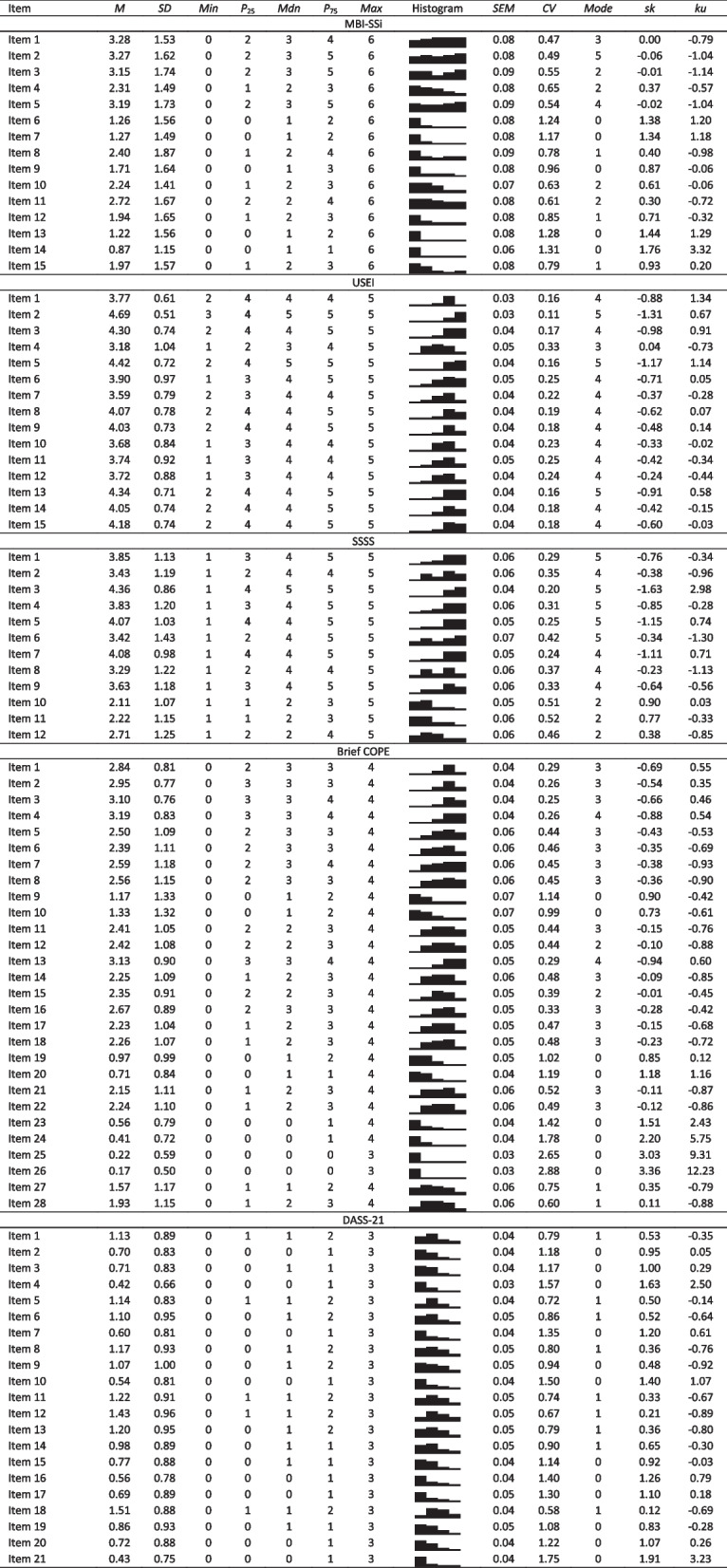
MBI-SSi, USEI, SSSS, Brief COPE, and DASS-21 descriptive statistics. Through the histograms, it is possible to visualize the distribution of answers by each item

##### Dimensionality

The original dimensionality of measuring instruments was verified using CFA to evaluate the data’s fit to the complete measurement model. The student burnout and academic engagement latent interaction variable was operationalized with three manifest variables from the MBI-SSi and three from the USEI. In order to avoid residuals’ negative variances, in six Brief-COPE dimensions (planning, religion, self-blame, venting, substance use, and humor) each pai r of items had their factor loadings constrained to be equal. The residual variance of USEI’s emotional engagement first-order factor was fixed to 0.01 to avoid negative variance. The measurement model presented a satisfactory fit to the data (χ^2^_(4,236__)_= 13,308.265; *p*<.001; χ^2^/*df*=3.142; *n*=389; *CFI*= . 949; *NFI*= .927; *TLI*= .948; *SRMR*= .088; *RMSEA*= .074; *P*(RMSEA≤ .05)< .001; 90%CI ] .073; .076[).

##### Reliability

The reliability of the scores in terms of internal consistency was estimated. Regarding the second-order latent factors, the internal consistency estimates were satisfactory at large: student burnout (ω_*L2*_ =  .891; ω_*L1*_ =  .865; ω_*partial L1*_ =  .955), academic engagement (ω_*L2*_ =  .893; ω_*L1*_ =  .742; ω_*partial L1*_ =  .863), satisfaction with social support (ω_*L2*_ =  .785; ω_*L1*_ =  .713; ω_*partial L1*_ =  .888), adaptive coping (ω_*L2*_ =  .783; ω_*L1*_ =  .753; ω_*partial L1*_ = .942), maladaptive coping (ω_*L2*_ =  .618; ω_*L1*_ =  .573; ω_*partial L1*_ =  .902) and general distress (ω_*L2*_ =  .922; ω_*L1*_ =  .883; ω_*partial L1*_ =  .958). The latent interaction factor presented an internal consistency estimate below the desirable (*ω* =  .581).

### Structural Model

**Fig. 2:**
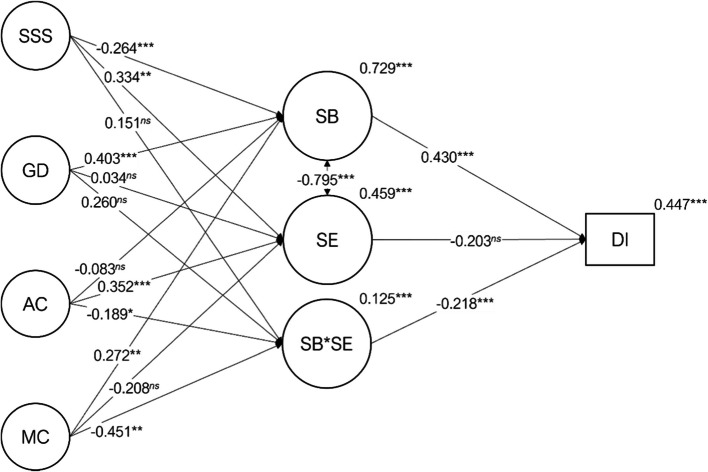
Path diagram with the regression paths, their statistical significance and *r*^2^ for the endogenous variables. SSS=Satisfaction with Social Sup port, AC=Adaptive Coping, MC=Maladaptive Coping, GD=General Distress, SB=Student Burnout, SE=Academic Engagement, SB*SE=Student Burnout interaction with Academic Engagement, DI=Dropout Intention. *ns* – *p*>.05; * – *p*≤.05; ** – *p*<.01; *** – *p*<.001

The latent variable structural model presented a good fit to the data (Fig. 2; χ^2^_(4, 32, 9)_=13,484.143; *p*<.001; χ^2^/*df*=3.115; *n*=389; *CFI*= .949; *NFI*= .927; *TLI*= .948; *SRMR*= .088; *RMSEA=* .074; *P*(RMSEA≤ .05)< .001; 90%CI ] .072; .075[). The direct effects of social support satisfaction (β_*SB<−SSS*_=-0.264; *p*<.001), general distress (β_*S B<−GD*_ = 0.403; *p*<.001) and maladaptive coping (β_*SB<−MC*_ = 0.272; *p*=.005) on burnout were statistically significant. The direct effects of social support satisfaction (β_*SE<−SSS*_ = 0.334; *p*=.002) and adaptive coping (β_*SE<−AC*_ = 0.352; *p*<.001) on academic engagement, revealed statistically significant paths. The effect of both coping latent variables on the latent moderation between burnout and academic engagement were statistically significant (β_SB*SE<−AC_=-0.189; *p*=.014; β_SB*SE<−MC_=-0.451; *p*=.004). Both student burnout (β_*DI<−SB*_ = 0.430; *p*<.001) and the latent moderation burnout x academic engagement (β_DI<−SB*SE_=-0.218; *p*<.001) presented a statically significant effect on dropout intention. While academic engagement did not present a statistically significant effect on dropout intention, however, the effect size was not negligible (β_DI<−SB*SE_=-0.203; *p*=.085). Table 2 presents all regression paths with a 95% confidence interval. The variance of the endogenous variables explained by the model presented large to low effect sizes (*r*^*2*^_*SB*_= . 729; *r*^*2*^_*SE*_= .459; *r*^*2*^_SB*SE_= .125; *r*^*2*^_*DI*_= .447). Figure 2 illustrates the standardized structural coefficients and their significance (α =  .05).

**Table 2:** Structural model’s regression paths

Path	*B*	*SE*	*Z*	β	*p*	95% CI
Student Burnout <- Satisfaction with Social Support	-0.349	0.094	-3.732	-0.265	< .001	]-0.533; -0.166[
Student Burnout <- General Distress	0.337	0.078	4.328	0.403	< .001	] 0.184; 0.489[
Student Burnout <- Adaptive Coping	-0.084	0.048	-1.761	-0.082	.078	]-0.177; 0.009[
Student Burnout <- Maladaptive Coping	0.352	0.120	2.922	0.272	.003	] 0.116; 0.588[
Academic Engagement <- Satisfaction with Social Support	0.278	0.088	3.157	0.334	.002	] 0.105; 0.450[
Academic Engagement <- General Distress	0.018	0.061	0.300	0.035	.764	]-0.101; 0.137[
Academic Engagement <- Adaptive Coping	0.225	0.048	4.666	0.352	< .001	] 0.131; 0.320[
Academic Engagement <- Maladaptive Coping	-0.169	0.091	-1.854	-0.208	.064	]-0.349; 0.010[
Interaction Student Burnout x Academic Engagement <- Satisfaction with Social Support	0.224	0.153	1.462	0.151	.144	]-0.076; 0.525[
Interaction Student Burnout x Academic Engagement <- General Distress	0.243	0.144	1.692	0.259	.091	]-0.039; 0.525[
Interaction Student Burnout x Academic Engagement <- Adaptive Coping	-0.216	0.088	-2.448	-0.189	.014	]-0.390; -0.043[
Interaction Student Burnout x Academic Engagement <- Maladaptive Coping	-0.655	0.230	-2.854	-0.451	.004	]-1.106; -0.205[
Dropout Intention <- Student Burnout	0.626	0.160	3.914	0.430	< .001	] 0.313; 0.939[
Dropout Intention <- Academic Engagement	-0.470	0.273	-1.721	-0.203	.085	]-1.006; 0.065[
Dropout Intention <- Interaction Student Burnout × Academic Engagement	-0.282	0.075	-3.772	-0.218	< .001	]-0.428; -0.135[

## Discussion

This study adds to medical education literature as it was the first time this structural model was applied to medical students. It showed that the higher the social support satisfaction, adaptive coping mechanism s, and academic engagement, the smaller the dropout intention. Academic engagement attenuates the impact of burnout on dropout intention, working as a protective factor. Also, it demonstrated that the higher the maladaptive coping mechanisms, general distress, and burnout, the higher the dropout intention. Additionally, every participant had the chance to reflect on the report of their answers and compare them with the overview, thus being able to take action if they had alarming results.

The model successfully allowed to verify the established hypothesis. The H1, partially confirmed, showed that adaptive coping did not significantly affect burnout. In contrast, social support satisfaction, general distress, and maladaptive coping presented a significant positive relation to burnout. It shows that the lack of adequate social support, depression, anxiety, stress, and not functional coping strategies are associated with higher levels of burnout. These findings highlight both the importance of peers in the academic path of medical students and the harmful impact that maladaptive behaviors can have on students’ mental health.

Adaptive coping strategies did not seem to be quite essential to prevent burnout. However, adaptive coping presented a meaningful relationship with academic engagement, as did social support satisfaction. Maladaptive coping and general distress did not show significant association, which allows to partially confirm H2. This fact underlines an exciting tendency as positive coping strategies are related to a positive construct (i.e., academic engagement). In contrast, maladaptive coping strategies are associated with a negative construct (i.e., student burnout). Social support satisfaction as burnout and academic engagement predictor presents a statistically significant relationship with both.

In this study, the direction of social support satisfaction to burnout was negative, while social support satisfaction to academic engagement was positive. Therefore, social support satisfaction can directly prevent burnout and promote academic engagement. Such finding emphasizes the substantial role that family, friends, and other peers have in medical students (particularly in the first year). It is known social support seems to have a solid association with student burnout [20] Nevertheless such association might depend on cultural factors, as the findings by Marôco et al. [5] suggest, where satisfaction with social support presented statistically significant relation with academic engagement and/or student burnout in some countries (e.g. Brazil, UK), while in others, no (e.g. Finland, Mozambique).

Depression-specific symptoms include dysphoria, discouragement, devaluation of life, self-deprecation, lack of interest or involvement, anhedonia, and inertia [45]. Stress may be beneficial in moderate levels, but the likelihood of illness is more significant in extreme levels [46]. Distress is the set of difficulty in relaxing, nervous excitement, easily agitated, overreaction, and impatience [45]. The general distress construct positively predicted burnout, as the model [47] posited it, but it did not present a meaningful effect on academic engagement. A negative relationship was expected; that is, the higher the distress, the lower the academic engagement. Moreover, the path from general distress and the interaction of burnout and academic engagement is significant. Nonetheless, this result deserves a more profound explanation in future studies. The role of general distress as a predictor is not clear because, in the absence of a longitudinal approach, it is impossible to know if it is a cause or an effect of burnout. All these relationships are multifactorial, and it is not easy to detect the main predictors to be intervened, and future investigation should focus on these main conclusions through longitudinal studies.

Burnout is a phenomenon that medical students face through college and later in their professional life. It is associated with risk factors such as maladaptive coping, general distress, and protective factors like social support satisfaction, adaptive coping, and academic engagement. Besides confirming this, this study demonstrated that these factors indirectly influence dropout intention. Academic engagement correlates to attending classes, submitting asked essays, and following teachers’ recommendations in class [13]. Burnout and engagement are negatively associated concepts as both can either be a cau se or a consequence of each other [5].

Burnout’s direct effect on dropout intention was statistically significant, confirming H3. However, academic engagement direct effect on dropout intention was not statistically significant, not confirming H4. Even though the regression path has the expected negative direction with a considerable magnitude and a statistical significance close to α (β = -0.203; *p* = .085).The observed findings (i.e. absence of a statistically significant effect) are identical to the results obtained by Marôco et al. [5] in a sample of eight countries and regions. Nevertheless, the moderation between academic engagement and burnout showed a meaningful path as expected (H5) [5]. Higher levels of burnout were related to higher dropout intention levels. The interaction of burnout and academic engagement represents the conjugated effect of these two variables, which indicated that academic engagement attenuates the effect of burnout (or vice-versa) on the dropout intention.

Despite the stress one is submitted to, it also comes to the response to tackle its source. On the one hand, people who tend to attribute an external locus of control prefer avoidant maladaptive strategies, which might show a higher tendency to develop burnout and disengagement [5]. On the other hand, people who claim outcomes result from one’s efforts tend to use adaptive coping strategies, which may have less tendency to develop burnout and disengagement. In this study, it was found that the path of maladaptive coping to burnout was positive, as was the path of adaptive coping to academic engagement. This idea favors ensuring that medical students who do not drop out are genuinely committed to being a doctor [48]. Also, one might assume that avoidance of maladaptive coping can be a focal point of intervention in preventing burnout and dropout intention. Whereas the positive path of adaptive coping to academic engagement, objected in this study, can predict that the development of adaptive coping directly influences academic engagement, which in turn will negatively impact burnout and dropout intention.

Burnout has educational and economic consequences, as it is associated with dropout intention [9]. Dropout is an indicator of the universities’ quality [49], and European Commission set the proportion of early leavers from education and training (the 18–24 year-olds) to be below 10% for Europe in 2020. However, it stood at 10.2% in 2019 [8]. Unfortunately, Portugal’s 2019 overview still has a worrying dropout rate in public universities [4]. Dropouts have costs for the individual and universities because the lower the number of students and graduates, the lower the financing received by universities [4]. Besides social-economic consequences of dropout, it is associated with personal seque ls, such as emotions of personal inadequacy, social stigm a, self-doubts, and ultimately a waste of time and resources [50]. Despite these facts, it should not be aimed to reduce the medical school dropo ut rate to 0% if it means postponing the dropout as doctors [48] or unprofessional behavior. Dyrbye et al. [51] described the association between unprofessional conduct and student burnout, as well as less altruistic values in medical students with burnout. Moreover, it is crucial to understand how students who suffer from burnout in college are more predisposed to develop job burnout [2], as heart disease, impaired cognitive performance, and other health conditions are associated with job burnout. Also, healthcare professionals with burnout showed more tendency to commit errors in the workplace, and compromised patients’ health resulted [52]. Considering future doctors’ performance link to healthcare conditions and quality of care in general, the goal is to prevent medical students’ tendency to job burnout acting primarily during their studies. To accomplish this, it means developing approaches to intensify social support satisf action of students, promoting their adaptive coping mechanisms, diminishing general distress and maladaptive coping strategies, which will promote academic engagement, and tackle burnout and dropout intention.

Ultimately, this study helps medical educators to guide them through tackling burnout and dropout in medical schools. Strategies that could implement a healthier individual status preventing burnout and dropout intention in medical schools are, for instance: a student support office that helps integrate students, especially the displaced ones, upgrading their social support satisfaction, having a psychology-psychiatric office that makes early diagnosis and treatment of depression and anxiety, and having workshops about preferring adaptive coping instead of maladaptive strategies. Longitudinal experimental studies should focus on the main results.

### Limitations

The critical weakness is the non-longitudinal approach of this study. It allows the study of correlations and must not necessarily lead to a causation interpretation as longitudinal and experimental studies would do. The lack of certainty regarding both the stability of the measures and the control of the process of potential generation of burnout drastically decreases the study’s internal validity.

Also, the data were collected during the SARS-CoV-2 pandemic and non-random sampling. As a result, the conclusions might have been affected by the context lived in Portugal. At the beginning of the academic year, as a period away from the exams, it is assumed that there should be no exacerbation effects. However, considering that the sample has first-year medical students, there may be adverse effects due to incorporation into the University environment.

Improvement of the comprehensibility and length of the survey, development of an individual report with overview comparison, the collection and analysis processes helped to make the sample more representative, which contributes to higher internal validity. However, the sample contains only 1st and 2nd-year students of a 6-years-course. Each year’s sample was insufficient and did not allow multigroup analysis (i.e., multigroup structural equation modeling). Also, this study was conducted in only one medical school of one country. Future research should study the application of this model and comparison in different universities and countries, therefore assessing the external validity of these results. If similar results are obtained, medical schools should apply longitudinal prospective studies with the implementation of supportive strategies of promotion of social support, adaptive coping and academic engagement, and prevention of maladaptive coping, general distress, burnout, and dropout intention.

## Conclusions

All in all, this cross-sectional study confirmed that burnout is a reality in Portuguese Medical students and that this structural model applies to these students and the relationship between variables was as expected. Regarding dropout intention, the relationship of social support satisfaction and adaptive coping is negative, while maladaptive coping and general distress is positive.

Considering that this country has a high need for doctors in the national health service, medical schools should implement strategies that focus on these psychological predictors and promote academic engagement to diminish burnout and dropout intention.

Future investigation should elaborate longitudinal and experimental studies to understand and tackle burnout and dropout intention. There is a lack of understanding on which interventions have the best results. In the future, the focus should be on these predictors and test prevention strategies of burnout and dropout rates and their consequences in medical schools.

## Data Availability

All of the material is owned by the authors and/or no permissions are required. The results/data/figures in this manuscript have not been published elsewhere, nor are they under consideration by another publisher. The datasets used and/or analysed during the current study are available from the corresponding author on reasonable request.
