# Correction: Burnout and dropout intention in medical students: the protective role of academic engagement

**DOI:** 10.1186/s12909-025-07924-y

**Published:** 2025-09-26

**Authors:** Sara Calcatin, Jorge Sinval, Lia Lucas Neto, João Marôco, António Gonçalves Ferreira, Pedro Oliveira

**Affiliations:** 1 Faculdade de Medicina, Universidade de Lisboa, Lisbon, Portugal; 2 Business Research Unit (BRU‑IUL), Instituto Universitario de Lisboa (ISCTE-IUL), Lisbon, Portugal; 3 William James Center for Research, ISPA - Instituto Universitario, Lisbon, Portugal; 4 Faculty of Philosophy, Sciences and Languages of Ribeirao Preto, University of Sao Paulo, Ribeirao Preto, SP Brazil; 5 CiiEM Centro investigacao interdisciplinar Egas Moniz, Almada, Portugal

**Correction: BMC Med Educ 22, 83 (2022)**

**https://doi.org/10.1186/s12909-021-03094-9**

Following publication of the original article [1], the authors requested an author name change of Sara Calcatin.

The incorrect author name is Sara Abreu Alves.

The correct author name is Sara Calcatin.

The author group has been updated above and the original article [1] has been corrected.
